# From image reconstruction to clinical prognosis: A comprehensive analysis of centiloid-based amyloid PET assessment

**DOI:** 10.1007/s00259-026-07943-x

**Published:** 2026-08-01

**Authors:** Katharina C. E. Hirschmüller, Elena Prieto, Edgar F. Guillén, Beatriz Echeveste, Fernando Mínguez, Marta Romera, Félix Pareja, Rachid Fahmi, Mario Riverol, Javier Arbizu

**Affiliations:** 1 Department of Nuclear Medicine, Clínica Universidad de Navarra, Pamplona, Spain; 2 Department of Medical Physics, Clínica Universidad de Navarra, 31008 Pamplona, Spain; 3 Department of Nuclear Medicine, Clínica Universidad de Navarra, Madrid, Spain; 4 Department of Neurology, Clínica Universidad de Navarra, Pamplona, Spain; 5 Molecular Imaging, Siemens Medical Solutions USA, Inc., Knoxville, TN USA

**Keywords:** Amyloid, PET, Centiloid, Alzheimer’s disease

## Abstract

**Purpose:**

To investigate the sources of variability in Centiloid (CL) calculations, particularly the influence of image reconstruction and reference region selection, and to examine the relationship between baseline CL scores, visual interpretation and subsequent disease progression.

**Methods:**

162 aMCI patients who underwent amyloid PET at a single center were retrospectively analyzed. Visual assessment was performed by two nuclear medicine physicians and Centiloid scoring was determined using syngo.MI Neurology Cortical Analysis, using different reference regions (RR) and image reconstruction settings. The CL values were compared against visual interpretation, using a ROC analysis. The value of CL in predicting the onset of Alzheimer's dementia was assessed.

**Results:**

The use of the whole cerebellum as RR provided the most robust and consistent CL values across reconstruction methods. The RR was critical in the case of flutemetamol, as CL varied in more than 20 units between pons and whole cerebellum. Visual classifications and CL values showed strong concordance (area under the ROC curve: 0.9786) and the CL cut-off value that maximized agreement with visual reading was 28 CL. During follow-up, 49% of patients progressed to AD dementia and CL-based amyloid positivity was a significant predictor of progression.

**Conclusion:**

Standardized CL quantification using the whole cerebellum as RR enhances the reliability of amyloid PET interpretation across tracers and reconstruction settings. CL values strongly correlate with visual assessment and are predictive of clinical progression. These findings suggest the potential utility of CL quantification in both clinical and research settings.

**Supplementary Information:**

The online version contains supplementary material available at https://doi.org/10.1007/s00259-026-07943-x.

## Introduction

Amyloid-β (Aβ) positron emission tomography (PET) enables in vivo visualization and quantification of Aβ deposition in the brain, a neuropathological hallmark of Alzheimer’s disease (AD) detectable decades before the manifestation of clinical symptoms. Aβ PET has become a validated biomarker in clinical trials, observational cohorts, and is increasingly used in clinical practice [1, 2]. Currently, three PET radiotracers, [^18^F]flutemetamol (FMM), [^18^F]florbetaben (FBB), and [^18^F]florbetapir (FBP) are approved by both by the U.S. Food and Drug Administration (FDA) and the European Medicines Agency (EMA) [3–5].

Structured image reading developed by manufacturers and approved by agencies relies on visual inspection by a physician and dichotomous classification (positive or negative). Additionally, quantitative analysis based on standardized uptake value ratios (SUVr) or Centiloids (CL) can support visual readings [6, 7]. SUVr is a tracer-specific ratio comparing uptake in cortical regions to a reference region (RR). Due to differences in tracer binding characteristics and reference region selection, SUVr are not directly comparable across tracers [8–10]. Centiloids are a rescaled version of SUVr on a 0–100 scale, with 0 representing the average amyloid level in young amyloid-negative controls and 100 the average level observed in typical AD patients [11]. Although the scale is anchored at these reference points, individual measurements are not restricted to this range, and values below 0 or above 100 may occur due to inherent variability. Centiloids enable comparability across different tracers; however, conversion from SUVr to CL requires tracer- and pipeline-specific calibration.

The CL score is essential for amyloid targeting therapies, as a standardized metric for patient selection and monitoring. As therapies move to earlier disease stages, CL allows consistent tracking of amyloid levels and treatment effects across studies. The EMA now recognizes CL as a qualified biomarker for amyloid load [12]. However, harmonizing measurements is necessary for reliable interpretation in both trials and clinical practice.

The original CL pipeline proposed by Klunk et al*.* [11] uses SPM with MRI-based spatial normalization, but alternative pipelines with different normalization methods have since emerged [8, 13]. In this investigation, the tool syngo.MI Neurology Cortical Analysis developed by Siemens Healthineers was used. This study aimed to investigate the sources of variability in Centiloid (CL) calculations, particularly the influence of image reconstruction and reference region selection, and to examine the relationship between baseline CL scores, visual interpretation and subsequent disease progression.

## Materials and methods

### Population

Patients with amnestic mild cognitive impairment (aMCI) [14] evaluated at the Memory Unit of Clínica Universidad de Navarra between 2009 and 2019 who underwent amyloid-PET imaging in routine clinical practice were included. Participants were assessed by an experienced neurologist using a standardized diagnostic workup, including medical history, informant interview, neurological examination, laboratory tests, brain MRI, and neuropsychological assessment, including Mini-Mental State Examination (MMSE). APOE-ε4 status was recorded when available. Exclusion criteria included age > 85 years, depression, abnormal laboratory findings, medical or substance-related conditions affecting cognition, and MRI evidence of other causes of brain damage [15].

Participants were followed in the Memory Unit with periodic neurological and neuropsychological assessments. Progression to AD dementia was defined according to the 2011 NIA-AA criteria [16]. Patients were followed until confirmed progression or for a minimum of 6 months if no progression occurred.

All participants provided written informed consent for the use of their data in research, and the study protocol was approved by the Ethics Research Committee of the Universidad de Navarra.

### Image acquisition

Aβ PET imaging was performed either with FMM (n = 51), FBB (n = 21), or FBP (n = 90) on a Siemens Biograph mCT scanner following standard tracer-specific protocols [5, 17, 18]. PET data acquired during the tracer-specific acquisition time window were reconstructed as a single static image. Images were reconstructed for visual reading using OSEM with time-of-flight and and point-spread-function (PSF) correction, 3 iterations, 21 subsets, 2-mm Gaussian filter, zoom factor 2, and 400 × 400 matrix (PSFg2). For 123 patients (51 FMM, 16 FBB, 56 FBP), images were additionally reconstructed without PSF and with a 5-mm Gaussian filter to produce smoothed images (nonPSFg5). NonPSFg5 reconstructions could not be generated for 39 patients due to the unavailability of the raw data.

### PET visual assessment

Amyloid PET scans (with PSFg2 reconstruction as standard) were independently assessed by two board-certified nuclear medicine physicians. Images were reviewed using the syngo.via platform without additional post-reconstruction image processing. Each scan was classified as either positive (V +) or negative (V-) based on visual uptake patterns following the respective radiotracer’s manufacturer guidelines. Cohen’s kappa was calculated to determine the level of agreement between readers. Discordant cases between readers were resolved by consensus during a joint review session.

### Centiloid quantification

Quantitative amyloid burden was determined using the syngo.MI Neurology Cortical Analysis solution (Siemens Medical Solutions USA, Inc.), a fully automated tool that is CE-marked and received FDA clearance in 2024 for Centiloid calculation in the clinical setting [19]. It should be noted that regulatory clearance pertains to the software's suitability for clinical use and does not constitute scientific validation of the quantitative metric. Accordingly, the scientific performance of this automated pipeline was evaluated in the present study through direct comparison with expert visual reading and correlation with clinical outcomes. Notably, unlike other available methods, this software does not require a structural MRI scan and automatically performs spatial normalisation of the PET image to a standard space.

Centiloid values were automatically obtained (using appropriate calibration equation embedded in the software) using tracer-specific target and reference regions (i.e. the pons for FMM, the cerebellar grey matter (CbGM) for FBB, and the whole cerebellum (WCb) for FBP). These radiotracer-recommended RR are referred to as TrSpec. Additionally, the WCb was used as RR for FBB and FMM. Although user interaction is allowed to slightly adjust the final spatial normalisation, no such adjustments were used in this study to ensure reproducibility.

#### Variability due to reference region and reconstruction

Four each PET scan, four CL values were systematically evaluated: CL1 was obtained for TRSpec RR and nonPSFg5 reconstruction, CL2 for TrSpec and PSFg2 reconstruction, CL3 for WCb and nonPSFg5 reconstruction and CL4 for WCb and PSFg2 reconstruction. For FPB, only two CL values are derived as the TrSpec RR is WCb.

Variability across methods was assessed for each tracer using Lin’s Concordance Correlation Coefficient (CCC) and Bland–Altman plots. Methods were considered concordant when CCC exceeded 0.99, mean bias was near zero, and limits of agreement (LoA) were within ± 10 CL.

#### Concordance with visual read

A Receiver Operating Characteristic (ROC) analysis compared CL values with visual reading and the optimal CL cut-off point was defined by maximising Youden’s J index, a metric combining sensitivity and specificity—calculated as the sum of sensitivity and specificity minus one—and representing the maximum vertical distance between the ROC curve and the diagonal of no discrimination [20]. Patients were then classified as quantitatively positive (Q +) or negative (Q-) using this optimal threshold and the concordance with visual classification was assessed.

#### Progression to alzheimer`s disease dementia

Kaplan–Meier survival analysis was performed to visualize progression to AD dementia over time within the Q + and Q- groups. Univariate Cox proportional hazards models were first applied to assess the effect of Centiloid, baseline MMSE, and APOE-ε4 carriership on the risk of conversion. Variables showing potential prognostic value in an univariate analysis were then included in a multivariate Cox model.

#### Statistical analysis

Analyses were conducted using Stata 15.1 (StataCorp, Texas, USA). Normality was assessed with the Shapiro–Wilk test. Normally distributed variables are presented as mean ± SD and compared using unpaired t-tests, while categorical variables were compared with the χ^2^ test. p-values < 0.05 were considered statistically significant.

## Results

### Demographics and visual reading

The cohort included 162 patients (71.3 ± 6.2 years; 42.6% women). Initial PET visual assessment showed high inter-reader agreement (Cohen’s Kappa 0.89). After a joint review, 66.0% were classified as visually positive (V +; Table 1). No difference in age, sex and tracer distribution was observed across V + and V- groups, while V + patients had lower MMSE scores and were more frequently APOE-ε4 carriers.

**Table 1:** Demographic and clinical characteristics of patients

	All	Negative visual read	Positive visual read	p-value
**N**	162	55	107	
**Age (years)**	71.3 ± 6.2	71.9 ± 6.7	71.0 ± 5.9	0.392
**Sex, females (%)**	42.6%	36.4%	45.8%	0.250
**Tracer (%)**				
FBB	13.0%	16.4%	11.2%	0.074
FMM	31.5%	20.0%	37.4%	
FBP	55.6%	63.6%	51.4%	
**MMSE**	26.1 ± 2.4	26.7 ± 2.5	25.9 ± 2.3	< 0.05
**APOE – e4 (%)**				
Non-carrier	37%	55%	28%	< 0.05
Carrier	37%	16%	48%	
No info available	26%	16%	24%	

### Centiloid quantification

#### Variability due to reference region and reconstruction

**Fig. 1:**
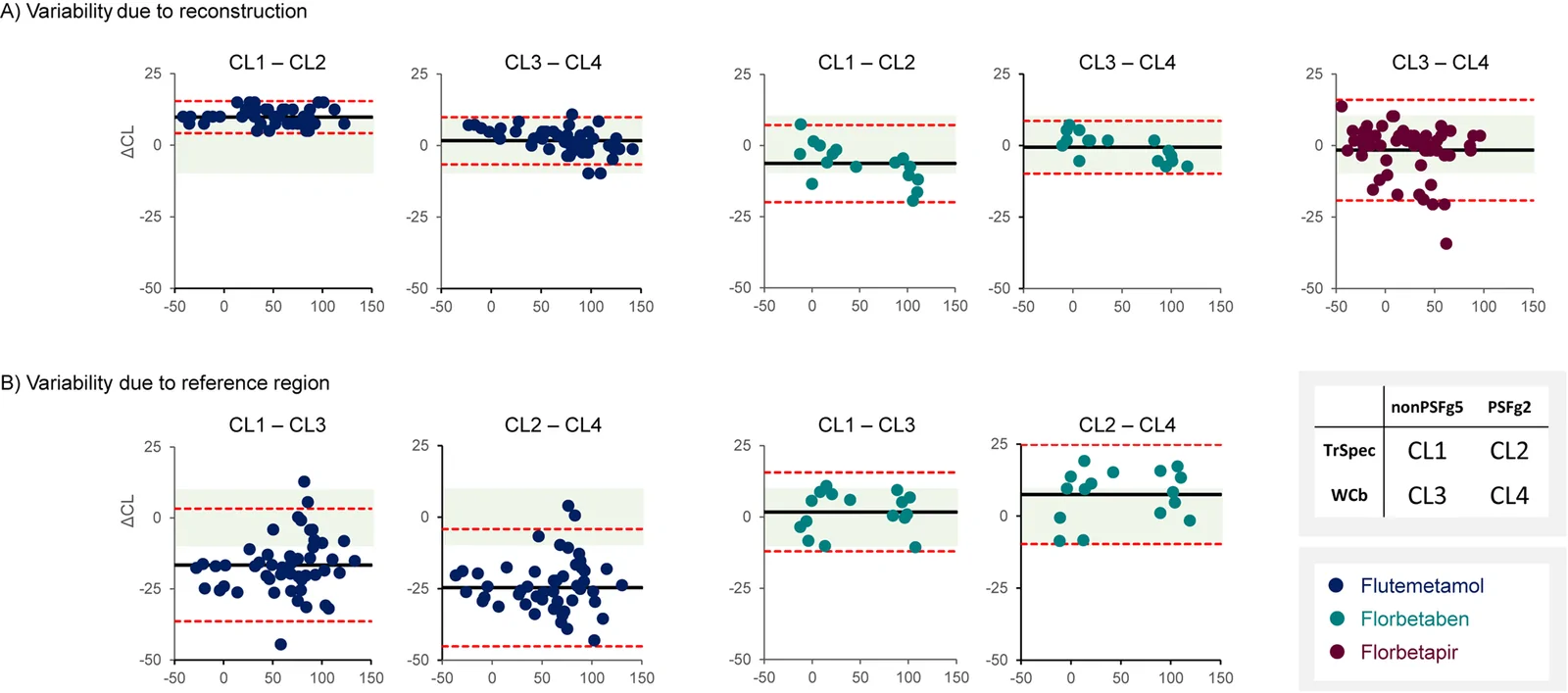
Bland–Altman plots showing the bias in CL relative to the mean CL across methods. A) Differences across reconstruction is presented while RR is fixed. B) The difference across RR is studied while reconstruction is fixed. The continuous black line represents the mean bias and the dashed red lines represent the 95% limits of agreement. Shaded green area, represent the area of tolerable variability of ± 10CL

Table 2 summarises the results of the assessment of variability in Centiloid values due to the two sources of variability considered: image reconstruction and RR. The four data obtained for each scan (CL1, CL2, CL3, and CL4) were compared by pairs, to analyse each source of variability (Fig. 1). The details of the Bland–Altman are presented on Table 2.

**Table 2:** Summary of Bland –Altman plot analysis of CL values changes with respect to reconstruction protocol and RR

**A Variability due to reconstruction**						
**Tracer**	**Comparison**	**Mean Bias (CL)**	**Lower LoA (CL)**	**Upper LoA (CL)**	**CCC**	**Concordance**
FMM	CL1 –CL2	9.8	4.2	15.4	0.968	Substantial
	CL3 –CL4	1.7	−6.6	9.9	0.993	Almost perfect
FBB	CL1 –CL2	−6.3	−19.9	7.2	0.982	Substantial
	CL3 –CL4	−0.6	−9.8	8.6	0.995	Almost perfect
FBP	CL3 –CL4	−1.6	−19.2	16.0	0.968	Substantial
**B Variability due to reference region**						
**Tracer**	**Comparison**	**Mean Bias**	**Lower LoA (CL)**	**Upper LoA (CL)**	**CCC**	**Concordance**
FMM	CL1-CL3	−16.6	−36.4	3.3	0.885	Poor
	CL2-CL4	−24.7	−45.2	−4.2	0.811	Poor
FBB	CL1-CL3	1.7	−12.1	15.6	0.988	Substantial
	CL2-CL4	7.5	−9.7	24.8	0.974	Substantial

The impact of reconstruction settings on CL values differs significantly across conditions (Fig. 1A). Concordance between reconstructions using WCb RR (CL3 vs. CL4) was almost perfect for both FMM and FBB, with a CCC > 0.99 and with 95% LoA within ± 10 CL. However, when TrSpec RR was used (CL1 vs. CL2), the CCC fell below 0.99 and the 95% LoA reached ± 20 CL. These results demonstrate the robustness of WCb against image reconstruction and, hence, this RR was selected for further analysis, across all three tracers. Note that FBP data show the highest variability with image reconstruction.

The choice of the RR has a significant impact on CL values (Fig. 1B). For FMM, concordance between pons- and WCb-derived CLs was poor (CCC ≤ 0.885), with a mean bias of 16.6 CL for nonPSFg5 and 24.7 CL for PSFg2 reconstruction. However, for FBB, the concordance of CL values obtained with different RR were much higher (CCC ≥ 0.974) and the bias closer to zero (i.e., −7.6 CL for nonPSFg5 and −2.1 CL for PSFg2). Therefore, in the case of FMM, the selection of the RR is critical. Following the recommendations in the existing literature [21, 22], we decided to disregard the use of pons and establish a unique reference region for all the tracers, namely whole cerebellum. Based on the results above, only CL values obtained with WCb as the RR using scans reconstructed with PSFg2 will be used for the rest of the analysis.

**Fig. 2:**
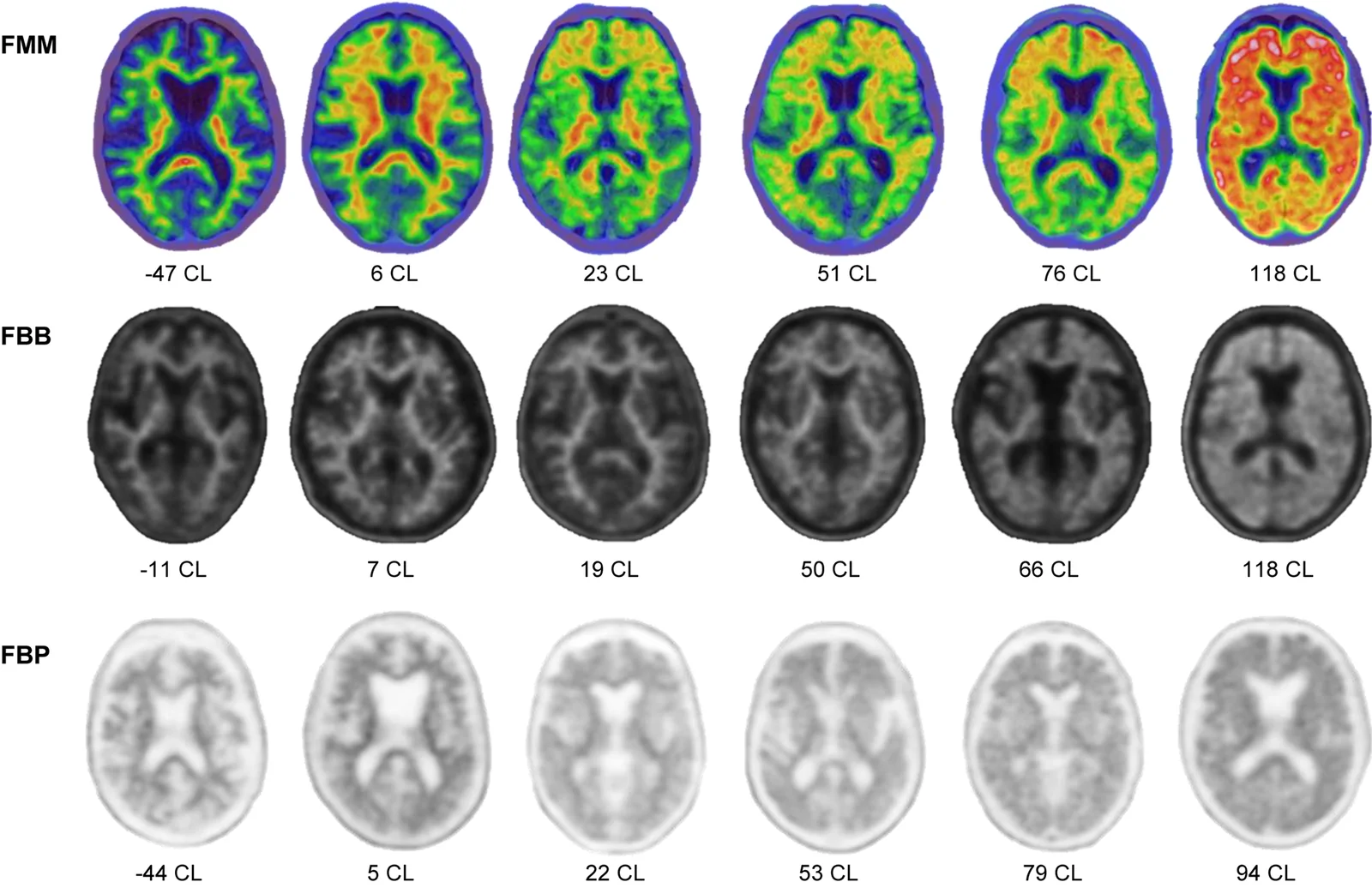
Exemplary slices of different amyloid PET scans with the respective Centiloids of each patient

Exemplary slices of the obtained PET images and their respective Centiloid values can be seen in Fig. 2. For each tracer, the color scale and intensity saturation were chosen according to the manufacturer’s guidelines.

#### Concordance with visual read

**Fig. 3:**
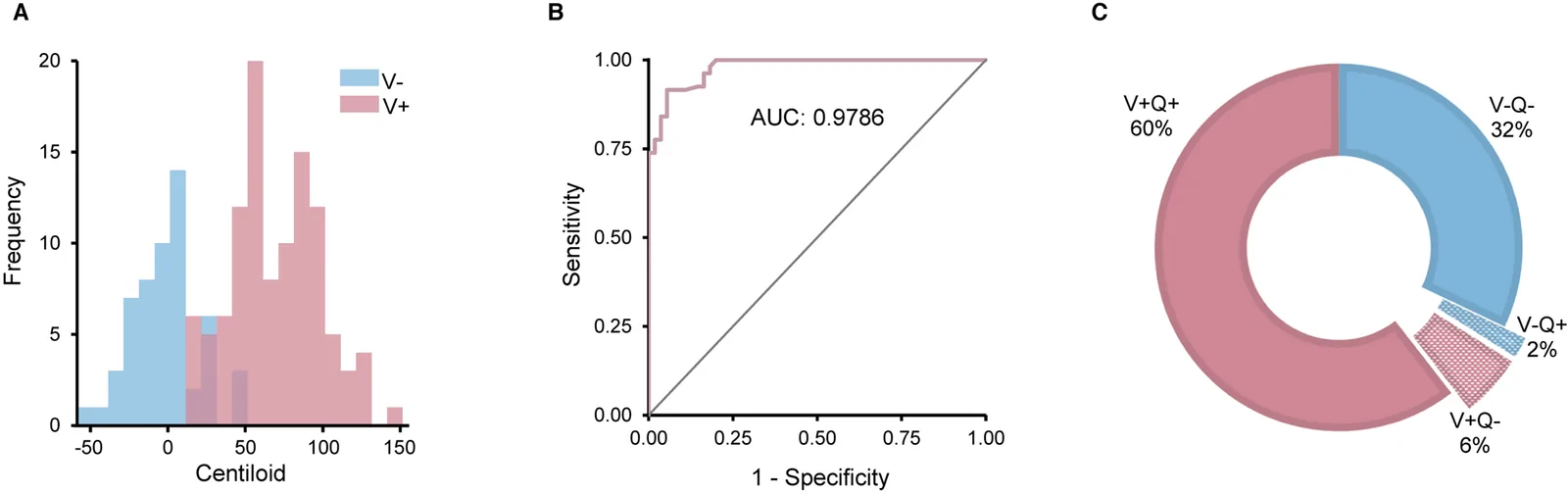
**A**) Histogram of CL values. **B**) ROC curve showing strong agreement between CL values and visual reading. **C**) Concordant and discordant cases between visual and quantitative classification

The group of visually positive (V +) patients exhibited a mean Centiloid value of 67.8 ± 29.2 CL, while the group of visually negative (V-) patients exhibited significantly lower Centiloids with a mean value of −3.0 ± 20.9 CL (Fig. 3, p < 0.05). Notably, an intermediate range of approximately 10 to 50 CL encompasses both visually positive and negative cases.

The ROC analysis yielded excellent agreement between visual-based classification and Centiloid values, with an area under the curve of 0.9786 (see Fig. 3). The CL cut-off value that maximized agreement with visual reading was 28 CL. At this cut-off point, concordant classification (i.e., V + Q + or V-Q-) was obtained in 92% of cases (Fig. 3). It is noteworthy that, for all four combinations of reconstruction and RR, the area under the ROC curve with visual reads ranged from 0.973 to 0.983.

#### Progression to alzheimer`s disease dementia

**Fig. 4:**
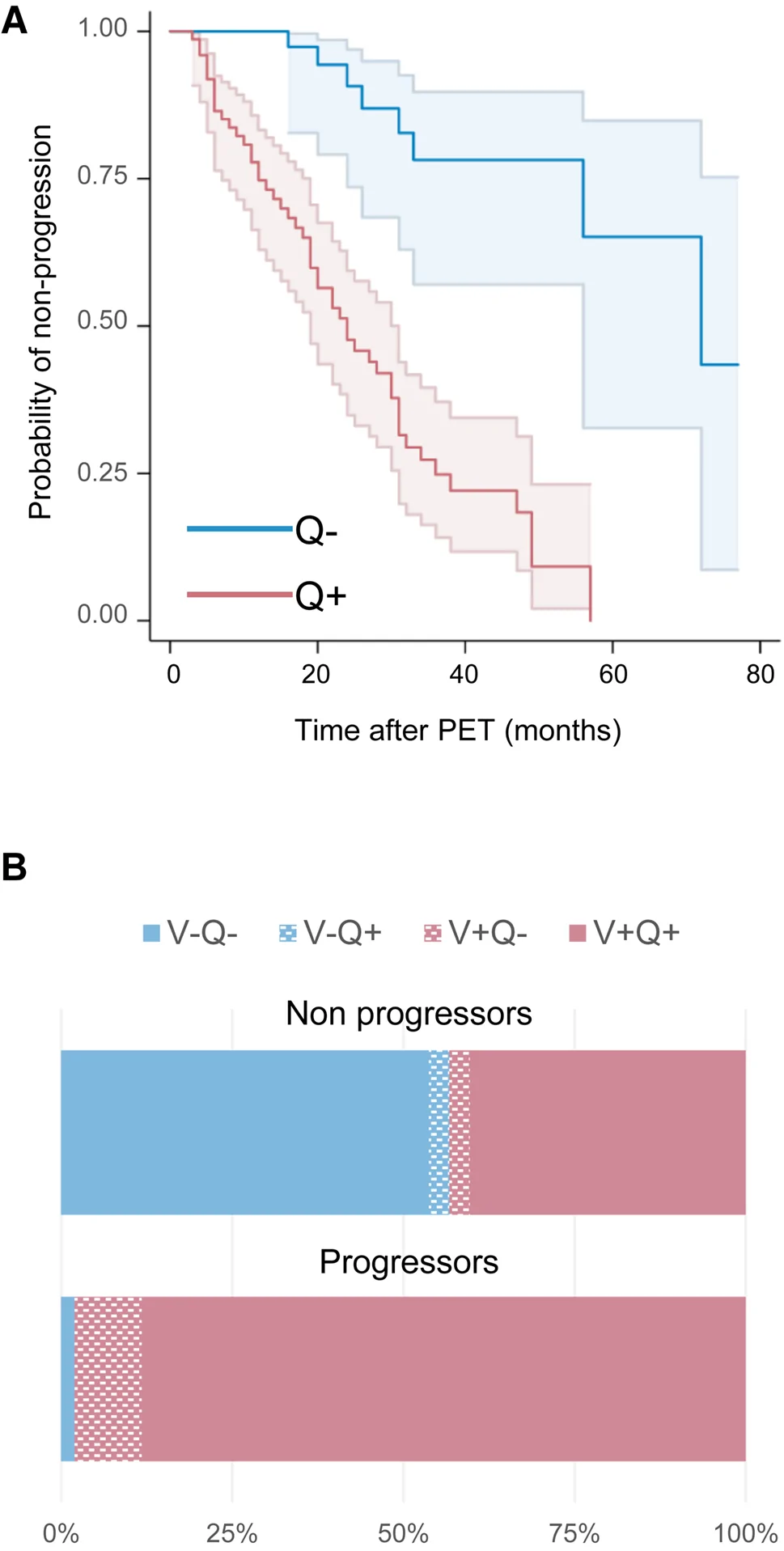
**A**) Kaplan–Meier survival curves comparing dementia progression to AD between patients with amyloid PET classified as Q + and Q-. (**B**) Distribution of the amyloid PET classifications for progressors and non-progresors

Clinical follow-up was available for 118 of 162 patients, of whom 58 (49%) progressed to AD. Kaplan–Meier survival curves (Fig. 4A) showed that Q + patients progressed significantly faster than Q- patients. Among progressors within 36 months, baseline PET was predominantly positive, whereas non-progressors included both positive and negative assessments (Fig. 4B). In univariate analyses, the Centiloid was significantly associated with progression (hazard ratio HR 1.03, 95% confidence interval CI: 1.02–1.03; p < 0.05), as well as APOE-ε4 carriership (HR 2.1, 95% CI: 1.1–4.0; p < 0.05) and baseline MMSE (HR 0.8, 95% CI: 0.8–0.9; p < 0.05). In the multivariate Cox proportional hazards model, only the Centiloid retained an independent prognostic value (HR 1.03, 95% CI: 1.02–1.04; p < 0.05).

## Discussion

Since its inception in 2015, the Centiloid scale developed by Klunk et al*.* [11] has undergone extensive validation in diverse settings [11, 23, 24]. In this single-centre retrospective study, we investigated the application of CL in a real-world series of images acquired using the three available amyloid PET tracers.

A key novel aspect of this study is the evaluation of the commercially available pipeline, syngo.MI Neurology Cortical Analysis. Unlike the standard CL method [11] which requires an MRI scan, CL calculation within syngo.MI Neurology Cortical Analysis is a PET-only pipeline, which is very practical as MRI data is not often available in clinical practice. Furthermore, this solution allows CL calculation using different RR. However, some of these RR are more sensitive to factors such as spatial normalization, image resolution and partial volume effects, and hence can result in different CL values for the same scan. In this study, we assessed the impact of RR selection and image reconstruction on CL values. This investigation may provide additional guidance on understanding the variability of this metric, particularly given that the increasing availability of software packages is making the Centiloid metric more accessible to facilities that are not primarily research-focused.

Although CL has been promoted as a universal metric providing comparable quantification across tracers and pipelines, it is important to understand that variability across PET machines and reconstruction settings remains an issue [25–27]. For this reason, much of the literature has focused on harmonising PET images for CL measurement [28–30]. In this study, all the images were acquired in the same PET system to avoid variability due to the tomograph. Therefore, the impact of different image resolution on CL evaluation was evaluated through image reconstruction. PSF modelling is generally discouraged for brain FDG scans due to artificial edge enhancement (Gibbs effect) between areas of abrupt change in uptake, as noted in EANM guidelines [31]. However, at our institution, PSF reconstruction is standard for amyloid imaging, as it reduces noise and improves contrast [32]. In our experience, the Gibbs effect is not visible on amyloid PET images due to low tracer uptake and low contrast between areas with and without binding. For this reason, we have included this reconstruction method in our quantification analysis. Our results show that different reconstructions can result in different CL values, in agreement with the results published by Zhang et al*.* [26], who demonstrated that image resolution impact Centiloids, especially in highly positive scans. In our population, mean differences between reconstructions were close to zero for FMM or FBP using WCb as RR (LoA ± 10 CL), with reconstruction type affecting FBP quantification more. Quantification in PET is influenced by partial volume effects (PVE) due to the modality’s limited spatial resolution. In particular, quantification of cortical amyloid uptake may be affected by spill-out from gray matter and spill-in from non-specific binding in adjacent white matter. Variations in retention among the three ^18^F-labeled amyloid tracers have been reported, which can alter gray-to-white matter contrast and, consequently, the balance between gray-matter spill-out and white-matter spill-in. Therefore, effective spatial resolution may impact Centiloid estimates, with the magnitude of this effect depending on the tracer and its specific retention characteristics [33]. We have decided to use the highest-resolution reconstruction, our visual analysis standard, supported by evidence that better resolution improves discrimination between positive and negative scans [29]. In this context, the EARL initiative promotes harmonized reconstruction, though specific guidelines for different tracers are still lacking [34]. The PSFg2 reconstruction used in this paper was requested for an EARL evaluation and resulted in an effective resolution of 4.5 mm, which is better than the EARL tolerance.

Regarding the reference region for CL assessment, our results show that CL may differ by more than 20 units in the case of FMM, with significantly lower pons-derived CL values, as observed in other studies [21, 22]. Such differences might impact, for example, eligibility decisions for amyloid targeting therapies as well as the assessment of response to therapy. Indeed, previous investigations have already discouraged the use of the pons as the reference region because it is primarily composed of white matter, and its amyloid uptake may depend on the age of the individual [11, 21, 22]. These results between CL values were replicated for the FMM dataset using another PET-only software (Supplementary Material). Hence, even though CL value can be obtained in syngo.MI Neurology Cortical Analysis directly with the usual tracer-specific regions used for SUVr calculations, we recommend changing the RR selection for CL derivation. Provided that the RR is left unchanged, the robustness of CL values across SPM pipelines [25] and even across commercial tools has been documented in earlier research [35–38]. Nevertheless, DiFilippo et al. [39] have found that individual CL scores often differ by more than 10 or even 20 CL between different software packages. Additionally, our results showed higher agreement between different RRs for nonPSFg5 than for PSFg2 image reconstruction.

In our cohort, we compared CL measurements to visual assessment and examined their relationship with clinical progression. The ROC analysis demonstrated that the CL scale performed well in discriminating between positive and negative amyloid subjects according to expert visual reading. This good discrimination was consistent for all methodological variants (RR and reconstruction). The optimal threshold derived from this analysis is consistent with previously published cut-off points [40–43]. However, using visual interpretation as the reference for determining thresholds and assessing performance may result in an optimistic estimate of real-world performance. This effect could be further amplified by the limited representation of patients in the ambiguous range from 10 to 50 CL associated with greater diagnostic uncertainty.

It should be noted that the cut-off point was primarily derived to demonstrate alignment with existing literature, rather than to establish a universal diagnostic threshold. In our series, for example, this cut-off threshold resulted in 8% of cases being discordant (V + Q- or V-Q +). Similarly, in the IDEAS study involving over 10,000 participants [44], 8% of scans were V + Q- and 6% were V-Q +, with discordant cases distributed across the entire Centiloid scale and associated with low-confidence visual readings. For this reason, binarisation can be reductive and we would like to advocate for the use of the Centiloid scale as a continuous variable as this approach more accurately reflects the biological reality of amyloid deposition.

In addition, our findings proved that, in both univariant and multivariate analyses, Centiloid score is a reliable indicator of progression, with each unit increase in CL raising the risk of progression (HR 1.03). These results are consistent with previous studies [41] and reinforce the importance of the quantitative score over a simple binary classification. Furthermore, multivariate analysis demonstrated that the quantitative Centiloid score has prognostic value independently of APOE-ε4 carriership and baseline MMSE. With regard to APOE-ε4, it has previously been reported that it influences early Aβ accumulation but has a limited impact in later AD stages [45–47]. Additionally, Bollack et al. [48] highlighted that APOE-ε4 carriership significantly impacts Aβ accumulation; however, this may not be generalised to cohorts consisting primarily of cognitively impaired individuals.

The main limitation of this study is the relatively small sample size of our cohort with uneven representation of the three amyloid PET tracers. Additionally, the cohort was composed solely of individuals with aMCI, representing a singular stage within the Alzheimer’s disease continuum. Thus, the extension of our results to preclinical or Alzheimer’s dementia stages remains uncertain. Despite these limitations, the present study provides evidence supporting the utilisation of automated software to quantify amyloid PET imaging in the clinical setting.

## Conclusion

The Centiloid scale is becoming increasingly accepted for Aβ-PET quantification in clinical routine, but its variability remains an issue. Our results showed that the selection of suitable reference regions is critical with the whole cerebellum being the most robust to image reconstruction. The impact of spatial resolution on the CL scale varies across tracers and that should be taken into account for reliable interpretation. Centiloid-based quantification showed good concordance with both visual binary classification and patient outcome. However, considering the scale as a continuous variable might be more suitable for tracking the progression of Alzheimer's disease and monitoring treatment response.

## Supplementary Information

Below is the link to the electronic supplementary material.

Supplementary file1 (DOCX 354 KB)

## Data Availability

The datasets generated during and/or analysed during the current study are available from the corresponding author on reasonable request.
