# Supplementary material for: From image reconstruction to clinical prognosis: A comprehensive analysis of centiloid-based amyloid PET assessment
Source: Eur J Nucl Med Mol Imaging. 2026 Aug 1;53(12):7538–47. doi: 10.1007/s00259-026-07943-x (PMC13633294; doi:10.1007/s00259-026-07943-x)
Supplement: Supplementary file 1 [file 259_2026_7943_MOESM1_ESM.docx]

Results Replication using rPoP Software

The impact of image reconstruction and that of reference regions on Centiloid values was also assessed using, rPoP [8], a well-established open source PET-based software that also involves non-linear warping of the input PET image to MNI space but uses GAAIN regions instead. Note that rPoP uses a smoothing step of the input image to a target isotropic resolution of FWHM=10mm^3^.

We focused on flutemetamol cases and compared Centiloid values obtained with the pons and WCb reference regions. As can be seen on Fig.1 and Fig. 2 below, similar correlation results between pons and WCb CL values were obtained using both SW solutions regardless of reconstruction method.

Figure 3 directly compares CL values calculated with the each of the SW packages using different reference regions and image reconstructions. The shown scatter plots show strong correlations between the two methods.

Comparison Pons vs Wcb (nonPSF)

CL_pons

| CL_Wcb | nonPSF - syngo |
| --- | --- |
| -19.1 | -36.678 |
| -6.9 | -31.679 |
| -13 | -29.1795 |
| 8.8 | -16.682 |
| 0.3 | -16.682 |
| 12.4 | -11.683 |
| 10 | -6.684 |
| 27 | 0.8145 |
| 1.5 | 3.314 |
| 31.8 | 20.8105 |
| 40.3 | 23.31 |
| 44 | 28.309 |
| 53.7 | 33.308 |
| 51.2 | 38.307 |
| 64.6 | 38.307 |
| 80.3 | 35.8075 |
| 57.3 | 35.8075 |
| 57.3 | 40.8065 |
| 68.2 | 48.305 |
| 68.2 | 50.8045 |
| 52.4 | 48.305 |
| 74.3 | 55.8035 |
| 81.5 | 55.8035 |
| 77.9 | 58.303 |
| 74.3 | 60.8025 |
| 90 | 60.8025 |
| 76.7 | 60.8025 |
| 86.4 | 65.8015 |
| 91.2 | 65.8015 |
| 90 | 68.301 |
| 99.7 | 68.301 |
| 82.7 | 68.301 |
| 75.5 | 75.7995 |
| 93.6 | 73.3 |
| 79.1 | 78.299 |
| 94.9 | 80.7985 |
| 103.3 | 83.298 |
| 90 | 85.7975 |
| 75.5 | 88.297 |
| 96.1 | 85.7975 |
| 119.1 | 88.297 |
| 92.4 | 88.297 |
| 96.1 | 88.297 |
| 111.8 | 93.296 |
| 122.7 | 90.7965 |
| 82.7 | 88.297 |
| 92.4 | 88.297 |
| 117.9 | 103.294 |
| 104.6 | 95.7955 |
| 127.6 | 108.293 |
| 126.4 | 118.291 |
| 140.9 | 125.79 |

| CL_Wcb | nonPSF - rPOP |
| --- | --- |
| -16.5878 | -32.1848 |
| -3.1005 | -29.711 |
| -15.516 | -36.4598 |
| -0.9185 | -18.8313 |
| -3.394 | -26.949 |
| -2.0414 | -22.8902 |
| -1.4544 | -14.4842 |
| 24.0911 | -2.932 |
| 10.2592 | 4.4652 |
| 26.809 | 20.3885 |
| 39.0841 | 21.3972 |
| 52.0865 | 40.1064 |
| 55.0341 | 35.327 |
| 58.2751 | 48.0801 |
| 66.6457 | 44.4775 |
| 82.9147 | 40.2986 |
| 53.3242 | 39.578 |
| 65.7908 | 41.6435 |
| 63.7364 | 57.6628 |
| 75.8329 | 57.3987 |
| 43.6649 | 34.0541 |
| 94.1435 | 84.0815 |
| 87.9294 | 59.8724 |
| 78.806 | 67.9181 |
| 80.3882 | 72.3612 |
| 98.8647 | 69.7674 |
| 80.3372 | 56.1258 |
| 77.0834 | 68.1102 |
| 91.0938 | 75.3153 |
| 89.116 | 75.6035 |
| 97.5376 | 75.0992 |
| 73.8678 | 69.4792 |
| 74.9142 | 92.4875 |
| 90.1879 | 79.038 |
| 91.6808 | 91.3827 |
| 110.042 | 99.6446 |
| 98.0225 | 81.0314 |
| 92.6378 | 91.2866 |
| 73.8296 | 82.6886 |
| 90.2772 | 89.2692 |
| 126.311 | 103.127 |
| 101.34 | 99.0441 |
| 84.7521 | 78.6297 |
| 95.6492 | 89.2692 |
| 116.537 | 99.0441 |
| 88.3249 | 101.614 |
| 110.247 | 105.361 |
| 104.9 | 106.922 |
| 95.7257 | 93.2801 |
| 132.487 | 117.489 |
| 127.932 | 129.257 |
| 155.06 | 146.934 |

Figure 1: comparison between pons and WCb Centiloid values calculated with either rPoP (red) or syngo.MI Neurology Cortical Analysis (blue) using nonPSF flutemetamol data (N=51).

Comparison Pons vs Wcb (PSF)

|  | CL WCb - TOF&PSF - syngo |
| --- | --- |
| -46.676 | -26.3 |
| -39.1775 | -13 |
| -39.1775 | -20.3 |
| -24.1805 | 5.2 |
| -24.1805 | -4.5 |
| -21.681 | 6.4 |
| -16.682 | 7.6 |
| -9.1835 | 22.1 |
| -4.1845 | -2.1 |
| 5.8135 | 23.4 |
| 13.312 | 40.3 |
| 15.8115 | 41.5 |
| 18.311 | 48.8 |
| 23.31 | 47.6 |
| 25.8095 | 59.7 |
| 25.8095 | 81.5 |
| 30.8085 | 58.5 |
| 33.308 | 52.4 |
| 35.8075 | 64.6 |
| 38.307 | 64.6 |
| 43.306 | 50 |
| 45.8055 | 77.9 |
| 48.305 | 74.3 |
| 48.305 | 74.3 |
| 50.8045 | 73 |
| 50.8045 | 87.6 |
| 50.8045 | 80.3 |
| 53.304 | 75.5 |
| 53.304 | 87.6 |
| 55.8035 | 88.8 |
| 55.8035 | 94.9 |
| 60.8025 | 81.5 |
| 63.302 | 73 |
| 65.8015 | 94.9 |
| 70.8005 | 81.5 |
| 73.3 | 97.3 |
| 75.7995 | 100.9 |
| 75.7995 | 92.4 |
| 78.299 | 74.3 |
| 80.7985 | 96.1 |
| 80.7985 | 123.9 |
| 80.7985 | 93.6 |
| 80.7985 | 98.5 |
| 80.7985 | 103.3 |
| 80.7985 | 123.9 |
| 83.298 | 82.7 |
| 83.298 | 102.1 |
| 88.297 | 117.9 |
| 88.297 | 114.3 |
| 93.296 | 128.8 |
| 105.793 | 123.9 |
| 118.291 | 142.1 |

|  |  |
| --- | --- |
| -43.2326 | -22.8912 |
| -36.9401 | -8.1152 |
| -42.488 | -18.6549 |
| -25.7722 | -3.713 |
| -31.9206 | -6.0863 |
| -29.3507 | -6.2394 |
| -24.5473 | -5.5376 |
| -9.1044 | 21.1052 |
| -1.2509 | 8.6004 |
| 10.5655 | 23.8997 |
| 16.1134 | 37.1446 |
| 32.493 | 51.474 |
| 25.7202 | 51.8186 |
| 42.34 | 58.0327 |
| 37.1523 | 65.3442 |
| 31.8926 | 80.7582 |
| 35.5912 | 52.4438 |
| 36.0475 | 65.6121 |
| 50.9381 | 62.6008 |
| 50.0975 | 75.1694 |
| 30.8118 | 43.9839 |
| 77.5009 | 93.531 |
| 55.117 | 87.151 |
| 62.4182 | 77.3513 |
| 65.6605 | 80.1585 |
| 66.7893 | 101.213 |
| 50.5538 | 79.9544 |
| 64.7719 | 78.0659 |
| 68.6866 | 90.9024 |
| 69.8154 | 90.6345 |
| 68.7827 | 99.2602 |
| 62.4903 | 70.9586 |
| 84.5138 | 73.3957 |
| 74.1385 | 93.8755 |
| 86.2671 | 92.0381 |
| 95.3695 | 112.748 |
| 78.8939 | 99.6941 |
| 86.6754 | 95.177 |
| 76.4201 | 72.6684 |
| 86.2911 | 91.8212 |
| 97.2669 | 128.493 |
| 94.0006 | 102.514 |
| 74.883 | 87.0106 |
| 85.4505 | 95.7768 |
| 96.3782 | 119.931 |
| 99.5485 | 91.7574 |
| 103.271 | 113.092 |
| 100.653 | 105.717 |
| 88.909 | 97.6014 |
| 111.821 | 133.916 |
| 125.799 | 131.198 |
| 146.334 | 161.44 |

Fig. 2: comparison between pons and WCb Centiloid values calculated with either rPoP (red) or syngo.MI Neurology Cortical Analysis (blue) using PSF flutemetamol data (N=51).

CL comparison: syngo.MI versus rPOP

rPOP

| syngo.MI Neurology | CL Pons - TOF - rPOP |
| --- | --- |
| -36.678 | -32.1848 |
| -31.679 | -29.711 |
| -29.1795 | -36.4598 |
| -16.682 | -18.8313 |
| -16.682 | -26.949 |
| -11.683 | -22.8902 |
| -6.684 | -14.4842 |
| 0.8145 | -2.932 |
| 3.314 | 4.4652 |
| 20.8105 | 20.3885 |
| 23.31 | 21.3972 |
| 28.309 | 40.1064 |
| 33.308 | 35.327 |
| 38.307 | 48.0801 |
| 38.307 | 44.4775 |
| 35.8075 | 40.2986 |
| 35.8075 | 39.578 |
| 40.8065 | 41.6435 |
| 48.305 | 57.6628 |
| 50.8045 | 57.3987 |
| 48.305 | 34.0541 |
| 55.8035 | 84.0815 |
| 55.8035 | 59.8724 |
| 58.303 | 67.9181 |
| 60.8025 | 72.3612 |
| 60.8025 | 69.7674 |
| 60.8025 | 56.1258 |
| 65.8015 | 68.1102 |
| 65.8015 | 75.3153 |
| 68.301 | 75.6035 |
| 68.301 | 75.0992 |
| 68.301 | 69.4792 |
| 75.7995 | 92.4875 |
| 73.3 | 79.038 |
| 78.299 | 91.3827 |
| 80.7985 | 99.6446 |
| 83.298 | 81.0314 |
| 85.7975 | 91.2866 |
| 88.297 | 82.6886 |
| 85.7975 | 89.2692 |
| 88.297 | 103.127 |
| 88.297 | 99.0441 |
| 88.297 | 78.6297 |
| 93.296 | 89.2692 |
| 90.7965 | 99.0441 |
| 88.297 | 101.614 |
| 88.297 | 105.361 |
| 103.294 | 106.922 |
| 95.7955 | 93.2801 |
| 108.293 | 117.489 |
| 118.291 | 129.257 |
| 125.79 | 146.934 |

| syngo.MI Neurology | CL Pons - TOF&PSF - rPOP |
| --- | --- |
| -46.676 | -43.2326 |
| -39.1775 | -36.9401 |
| -39.1775 | -42.488 |
| -24.1805 | -25.7722 |
| -24.1805 | -31.9206 |
| -21.681 | -29.3507 |
| -16.682 | -24.5473 |
| -9.1835 | -9.1044 |
| -4.1845 | -1.2509 |
| 5.8135 | 10.5655 |
| 13.312 | 16.1134 |
| 15.8115 | 32.493 |
| 18.311 | 25.7202 |
| 23.31 | 42.34 |
| 25.8095 | 37.1523 |
| 25.8095 | 31.8926 |
| 30.8085 | 35.5912 |
| 33.308 | 36.0475 |
| 35.8075 | 50.9381 |
| 38.307 | 50.0975 |
| 43.306 | 30.8118 |
| 45.8055 | 77.5009 |
| 48.305 | 55.117 |
| 48.305 | 62.4182 |
| 50.8045 | 65.6605 |
| 50.8045 | 66.7893 |
| 50.8045 | 50.5538 |
| 53.304 | 64.7719 |
| 53.304 | 68.6866 |
| 55.8035 | 69.8154 |
| 55.8035 | 68.7827 |
| 60.8025 | 62.4903 |
| 63.302 | 84.5138 |
| 65.8015 | 74.1385 |
| 70.8005 | 86.2671 |
| 73.3 | 95.3695 |
| 75.7995 | 78.8939 |
| 75.7995 | 86.6754 |
| 78.299 | 76.4201 |
| 80.7985 | 86.2911 |
| 80.7985 | 97.2669 |
| 80.7985 | 94.0006 |
| 80.7985 | 74.883 |
| 80.7985 | 85.4505 |
| 80.7985 | 96.3782 |
| 83.298 | 99.5485 |
| 83.298 | 103.271 |
| 88.297 | 100.653 |
| 88.297 | 88.909 |
| 93.296 | 111.821 |
| 105.793 | 125.799 |
| 118.291 | 146.334 |

| syngo.MI Neurology | CL WCb - TOF - rPOP |
| --- | --- |
| -19.1 | -16.5878 |
| -6.9 | -3.1005 |
| -13 | -15.516 |
| 8.8 | -0.9185 |
| 0.3 | -3.394 |
| 12.4 | -2.0414 |
| 10 | -1.4544 |
| 27 | 24.0911 |
| 1.5 | 10.2592 |
| 31.8 | 26.809 |
| 40.3 | 39.0841 |
| 44 | 52.0865 |
| 53.7 | 55.0341 |
| 51.2 | 58.2751 |
| 64.6 | 66.6457 |
| 80.3 | 82.9147 |
| 57.3 | 53.3242 |
| 57.3 | 65.7908 |
| 68.2 | 63.7364 |
| 68.2 | 75.8329 |
| 52.4 | 43.6649 |
| 74.3 | 94.1435 |
| 81.5 | 87.9294 |
| 77.9 | 78.806 |
| 74.3 | 80.3882 |
| 90 | 98.8647 |
| 76.7 | 80.3372 |
| 86.4 | 77.0834 |
| 91.2 | 91.0938 |
| 90 | 89.116 |
| 99.7 | 97.5376 |
| 82.7 | 73.8678 |
| 75.5 | 74.9142 |
| 93.6 | 90.1879 |
| 79.1 | 91.6808 |
| 94.9 | 110.042 |
| 103.3 | 98.0225 |
| 90 | 92.6378 |
| 75.5 | 73.8296 |
| 96.1 | 90.2772 |
| 119.1 | 126.311 |
| 92.4 | 101.34 |
| 96.1 | 84.7521 |
| 111.8 | 95.6492 |
| 122.7 | 116.537 |
| 82.7 | 88.3249 |
| 92.4 | 110.247 |
| 117.9 | 104.9 |
| 104.6 | 95.7257 |
| 127.6 | 132.487 |
| 126.4 | 127.932 |
| 140.9 | 155.06 |

| syngo.MI Neurology | CL WCb - TOF&PSF - rPOP |
| --- | --- |
| -26.3 | -22.8912 |
| -13 | -8.1152 |
| -20.3 | -18.6549 |
| 5.2 | -3.713 |
| -4.5 | -6.0863 |
| 6.4 | -6.2394 |
| 7.6 | -5.5376 |
| 22.1 | 21.1052 |
| -2.1 | 8.6004 |
| 23.4 | 23.8997 |
| 40.3 | 37.1446 |
| 41.5 | 51.474 |
| 48.8 | 51.8186 |
| 47.6 | 58.0327 |
| 59.7 | 65.3442 |
| 81.5 | 80.7582 |
| 58.5 | 52.4438 |
| 52.4 | 65.6121 |
| 64.6 | 62.6008 |
| 64.6 | 75.1694 |
| 50 | 43.9839 |
| 77.9 | 93.531 |
| 74.3 | 87.151 |
| 74.3 | 77.3513 |
| 73 | 80.1585 |
| 87.6 | 101.213 |
| 80.3 | 79.9544 |
| 75.5 | 78.0659 |
| 87.6 | 90.9024 |
| 88.8 | 90.6345 |
| 94.9 | 99.2602 |
| 81.5 | 70.9586 |
| 73 | 73.3957 |
| 94.9 | 93.8755 |
| 81.5 | 92.0381 |
| 97.3 | 112.748 |
| 100.9 | 99.6941 |
| 92.4 | 95.177 |
| 74.3 | 72.6684 |
| 96.1 | 91.8212 |
| 123.9 | 128.493 |
| 93.6 | 102.514 |
| 98.5 | 87.0106 |
| 103.3 | 95.7768 |
| 123.9 | 119.931 |
| 82.7 | 91.7574 |
| 102.1 | 113.092 |
| 117.9 | 105.717 |
| 114.3 | 97.6014 |
| 128.8 | 133.916 |
| 123.9 | 131.198 |
| 142.1 | 161.44 |

Figure 3: comparison between Centiloid values calculated with either rPoP and those calculated with syngo.MI Neurology Cortical Analysis using different reference regions and flutemetamol data (N=51) reconstructed either with or without PSF.

Testing the visual read against all combinations of Reference Region and Reconstruction

To further, assess the variability due to Reference Region (RR) and Reconstruction, we repeated the Receiver Operating Characteristic (ROC) analysis with all combinations of Reconstruction and RR and compared the CL values of each method with visual reading.

All four areas under the ROC Curve are shown in Table 1 and a combined plot of all four ROC curves can be seen in Figure 3. The areas under the curve between 0.973 and 0.983 indicate a good discrimination for all four methods. Additionally, the ROC curves exhibit a substantial degree of similarity.

Table 1: Areas under the ROC curve of the comparison between the visual read against CL values obtained with different RRs and reconstructions.

|  | NonPSFg5 | PSFg2 |
| --- | --- | --- |
| TrSpec | CL1  AUC = 0.9834  N = 123 | CL2  AUC = 0.9816  N = 162 |
| WCb | CL3  AUC = 0.9733  N = 123 | CL4  AUC = 0.9786  N = 162 |

|  | CL1 |
| --- | --- |
| 1 | 1 |
| 0.9744 | 1 |
| 0.9487 | 1 |
| 0.9231 | 1 |
| 0.8974 | 1 |
| 0.8462 | 1 |
| 0.8205 | 1 |
| 0.7949 | 1 |
| 0.7692 | 1 |
| 0.7179 | 1 |
| 0.6923 | 1 |
| 0.641 | 1 |
| 0.5897 | 1 |
| 0.5641 | 1 |
| 0.5385 | 1 |
| 0.5128 | 1 |
| 0.4872 | 1 |
| 0.4615 | 1 |
| 0.4359 | 1 |
| 0.4103 | 1 |
| 0.3846 | 1 |
| 0.359 | 1 |
| 0.3333 | 1 |
| 0.3077 | 1 |
| 0.2821 | 1 |
| 0.2564 | 1 |
| 0.2308 | 1 |
| 0.2308 | 0.9881 |
| 0.2308 | 0.9762 |
| 0.2051 | 0.9643 |
| 0.1795 | 0.9643 |
| 0.1538 | 0.9643 |
| 0.1538 | 0.9405 |
| 0.1282 | 0.9405 |
| 0.1282 | 0.9286 |
| 0.1026 | 0.9286 |
| 0.1026 | 0.9167 |
| 0.0769 | 0.9167 |
| 0.0513 | 0.9167 |
| 0.0513 | 0.9048 |
| 0.0256 | 0.9048 |
| 0.0256 | 0.8929 |
| 0.0256 | 0.869 |
| 0.0256 | 0.8571 |
| 0.0256 | 0.8452 |
| 0 | 0.8452 |
| 0 | 0.8214 |
| 0 | 0.7976 |
| 0 | 0.7738 |
| 0 | 0.75 |
| 0 | 0.7381 |
| 0 | 0.7262 |
| 0 | 0.7143 |
| 0 | 0.6905 |
| 0 | 0.6667 |
| 0 | 0.6429 |
| 0 | 0.619 |
| 0 | 0.6071 |
| 0 | 0.5952 |
| 0 | 0.5833 |
| 0 | 0.5714 |
| 0 | 0.5476 |
| 0 | 0.5357 |
| 0 | 0.5 |
| 0 | 0.4881 |
| 0 | 0.4524 |
| 0 | 0.4405 |
| 0 | 0.4167 |
| 0 | 0.381 |
| 0 | 0.369 |
| 0 | 0.3571 |
| 0 | 0.3452 |
| 0 | 0.3333 |
| 0 | 0.3214 |
| 0 | 0.3095 |
| 0 | 0.2976 |
| 0 | 0.2738 |
| 0 | 0.25 |
| 0 | 0.1786 |
| 0 | 0.1667 |
| 0 | 0.1548 |
| 0 | 0.1429 |
| 0 | 0.131 |
| 0 | 0.119 |
| 0 | 0.1071 |
| 0 | 0.0952 |
| 0 | 0.0833 |
| 0 | 0.0714 |
| 0 | 0.0595 |
| 0 | 0.0476 |
| 0 | 0.0357 |
| 0 | 0.0238 |
| 0 | 0.0119 |
| 0 | 0 |

|  | CL3 |
| --- | --- |
| 1 | 1 |
| 0.9744 | 1 |
| 0.9487 | 1 |
| 0.8974 | 1 |
| 0.8718 | 1 |
| 0.8462 | 1 |
| 0.7949 | 1 |
| 0.7692 | 1 |
| 0.7436 | 1 |
| 0.6923 | 1 |
| 0.6667 | 1 |
| 0.641 | 1 |
| 0.6154 | 1 |
| 0.5897 | 1 |
| 0.5641 | 1 |
| 0.5385 | 1 |
| 0.5128 | 1 |
| 0.4872 | 1 |
| 0.4615 | 1 |
| 0.4359 | 1 |
| 0.4103 | 1 |
| 0.3846 | 1 |
| 0.359 | 1 |
| 0.3333 | 1 |
| 0.3333 | 0.9881 |
| 0.3077 | 0.9881 |
| 0.2821 | 0.9881 |
| 0.2564 | 0.9881 |
| 0.2308 | 0.9881 |
| 0.2051 | 0.9762 |
| 0.1795 | 0.9762 |
| 0.1538 | 0.9762 |
| 0.1538 | 0.9524 |
| 0.1282 | 0.9524 |
| 0.1282 | 0.9405 |
| 0.1282 | 0.9286 |
| 0.1282 | 0.9167 |
| 0.1282 | 0.9048 |
| 0.1026 | 0.9048 |
| 0.0769 | 0.9048 |
| 0.0769 | 0.881 |
| 0.0769 | 0.869 |
| 0.0513 | 0.869 |
| 0.0513 | 0.8571 |
| 0.0513 | 0.8452 |
| 0.0513 | 0.8214 |
| 0.0513 | 0.7976 |
| 0.0256 | 0.7976 |
| 0.0256 | 0.7857 |
| 0.0256 | 0.7619 |
| 0.0256 | 0.75 |
| 0.0256 | 0.7262 |
| 0.0256 | 0.7024 |
| 0.0256 | 0.6905 |
| 0.0256 | 0.6786 |
| 0.0256 | 0.6667 |
| 0.0256 | 0.6548 |
| 0.0256 | 0.6429 |
| 0 | 0.6429 |
| 0 | 0.619 |
| 0 | 0.5833 |
| 0 | 0.5714 |
| 0 | 0.5595 |
| 0 | 0.5476 |
| 0 | 0.5238 |
| 0 | 0.5119 |
| 0 | 0.4881 |
| 0 | 0.4643 |
| 0 | 0.4524 |
| 0 | 0.4405 |
| 0 | 0.4286 |
| 0 | 0.4167 |
| 0 | 0.4048 |
| 0 | 0.381 |
| 0 | 0.3571 |
| 0 | 0.3333 |
| 0 | 0.3214 |
| 0 | 0.2857 |
| 0 | 0.2738 |
| 0 | 0.2619 |
| 0 | 0.25 |
| 0 | 0.2262 |
| 0 | 0.2143 |
| 0 | 0.2024 |
| 0 | 0.1786 |
| 0 | 0.1667 |
| 0 | 0.1548 |
| 0 | 0.131 |
| 0 | 0.119 |
| 0 | 0.1071 |
| 0 | 0.0952 |
| 0 | 0.0833 |
| 0 | 0.0714 |
| 0 | 0.0595 |
| 0 | 0.0476 |
| 0 | 0.0357 |
| 0 | 0.0238 |
| 0 | 0.0119 |
| 0 | 0 |

|  | CL2 |
| --- | --- |
| 1 | 1 |
| 0.9818 | 1 |
| 0.9636 | 1 |
| 0.9455 | 1 |
| 0.9091 | 1 |
| 0.8909 | 1 |
| 0.8727 | 1 |
| 0.8545 | 1 |
| 0.7818 | 1 |
| 0.7091 | 1 |
| 0.6545 | 1 |
| 0.6364 | 1 |
| 0.6 | 1 |
| 0.5818 | 1 |
| 0.5636 | 1 |
| 0.5455 | 1 |
| 0.5273 | 1 |
| 0.5091 | 1 |
| 0.4545 | 1 |
| 0.4182 | 1 |
| 0.4 | 1 |
| 0.3818 | 1 |
| 0.3636 | 1 |
| 0.3273 | 1 |
| 0.3091 | 1 |
| 0.2909 | 1 |
| 0.2182 | 1 |
| 0.2182 | 0.9907 |
| 0.2 | 0.972 |
| 0.1818 | 0.972 |
| 0.1818 | 0.9533 |
| 0.1636 | 0.9533 |
| 0.1636 | 0.9346 |
| 0.1455 | 0.9346 |
| 0.1273 | 0.9346 |
| 0.1273 | 0.9159 |
| 0.0727 | 0.9065 |
| 0.0545 | 0.8972 |
| 0.0364 | 0.8972 |
| 0.0182 | 0.8785 |
| 0.0182 | 0.8692 |
| 0.0182 | 0.8598 |
| 0.0182 | 0.8505 |
| 0.0182 | 0.8224 |
| 0.0182 | 0.8037 |
| 0.0182 | 0.7944 |
| 0 | 0.7944 |
| 0 | 0.7757 |
| 0 | 0.7664 |
| 0 | 0.7103 |
| 0 | 0.6822 |
| 0 | 0.6542 |
| 0 | 0.6355 |
| 0 | 0.6075 |
| 0 | 0.5981 |
| 0 | 0.5234 |
| 0 | 0.4953 |
| 0 | 0.4766 |
| 0 | 0.4673 |
| 0 | 0.4393 |
| 0 | 0.4299 |
| 0 | 0.4206 |
| 0 | 0.4112 |
| 0 | 0.4019 |
| 0 | 0.3832 |
| 0 | 0.3551 |
| 0 | 0.3458 |
| 0 | 0.3364 |
| 0 | 0.3271 |
| 0 | 0.3178 |
| 0 | 0.3084 |
| 0 | 0.2897 |
| 0 | 0.2804 |
| 0 | 0.271 |
| 0 | 0.215 |
| 0 | 0.1963 |
| 0 | 0.1869 |
| 0 | 0.1776 |
| 0 | 0.1402 |
| 0 | 0.1308 |
| 0 | 0.1215 |
| 0 | 0.1121 |
| 0 | 0.1028 |
| 0 | 0.0935 |
| 0 | 0.0841 |
| 0 | 0.0561 |
| 0 | 0.0467 |
| 0 | 0.028 |
| 0 | 0.0187 |
| 0 | 0.0093 |
| 0 | 0 |

|  | CL4 |
| --- | --- |
| 1 | 1 |
| 0.9818 | 1 |
| 0.9636 | 1 |
| 0.9455 | 1 |
| 0.9273 | 1 |
| 0.9091 | 1 |
| 0.8909 | 1 |
| 0.8545 | 1 |
| 0.8 | 1 |
| 0.7818 | 1 |
| 0.7455 | 1 |
| 0.7091 | 1 |
| 0.6909 | 1 |
| 0.6727 | 1 |
| 0.6545 | 1 |
| 0.6364 | 1 |
| 0.6182 | 1 |
| 0.6 | 1 |
| 0.5636 | 1 |
| 0.5091 | 1 |
| 0.4727 | 1 |
| 0.4545 | 1 |
| 0.4364 | 1 |
| 0.4182 | 1 |
| 0.3818 | 1 |
| 0.3636 | 1 |
| 0.3455 | 1 |
| 0.3273 | 1 |
| 0.3091 | 1 |
| 0.2909 | 1 |
| 0.2364 | 1 |
| 0.2182 | 1 |
| 0.2 | 1 |
| 0.1818 | 0.9813 |
| 0.1818 | 0.9626 |
| 0.1636 | 0.9626 |
| 0.1636 | 0.9533 |
| 0.1636 | 0.9439 |
| 0.1636 | 0.9252 |
| 0.1455 | 0.9252 |
| 0.1091 | 0.9159 |
| 0.0909 | 0.9159 |
| 0.0727 | 0.9159 |
| 0.0545 | 0.9159 |
| 0.0545 | 0.9065 |
| 0.0545 | 0.8972 |
| 0.0545 | 0.8785 |
| 0.0545 | 0.8692 |
| 0.0545 | 0.8598 |
| 0.0545 | 0.8411 |
| 0.0364 | 0.8411 |
| 0.0364 | 0.8318 |
| 0.0364 | 0.8224 |
| 0.0364 | 0.7757 |
| 0.0182 | 0.7757 |
| 0.0182 | 0.757 |
| 0.0182 | 0.7477 |
| 0.0182 | 0.7383 |
| 0 | 0.7383 |
| 0 | 0.729 |
| 0 | 0.7196 |
| 0 | 0.7103 |
| 0 | 0.7009 |
| 0 | 0.6449 |
| 0 | 0.6355 |
| 0 | 0.6075 |
| 0 | 0.5981 |
| 0 | 0.5888 |
| 0 | 0.5514 |
| 0 | 0.5421 |
| 0 | 0.5234 |
| 0 | 0.514 |
| 0 | 0.4953 |
| 0 | 0.4673 |
| 0 | 0.4579 |
| 0 | 0.4486 |
| 0 | 0.4299 |
| 0 | 0.4019 |
| 0 | 0.3925 |
| 0 | 0.3832 |
| 0 | 0.3738 |
| 0 | 0.3645 |
| 0 | 0.3364 |
| 0 | 0.3271 |
| 0 | 0.3178 |
| 0 | 0.3084 |
| 0 | 0.2991 |
| 0 | 0.2617 |
| 0 | 0.2523 |
| 0 | 0.243 |
| 0 | 0.2336 |
| 0 | 0.2243 |
| 0 | 0.215 |
| 0 | 0.2056 |
| 0 | 0.1869 |
| 0 | 0.1776 |
| 0 | 0.1682 |
| 0 | 0.1589 |
| 0 | 0.1495 |
| 0 | 0.1308 |
| 0 | 0.1215 |
| 0 | 0.1121 |
| 0 | 0.1028 |
| 0 | 0.0935 |
| 0 | 0.0841 |
| 0 | 0.0748 |
| 0 | 0.0654 |
| 0 | 0.0561 |
| 0 | 0.0467 |
| 0 | 0.0187 |
| 0 | 0.0093 |
| 0 | 0 |

|  |  |
| --- | --- |
| 0 | 0 |
| 1 | 1 |

Figure 4: ROC curve showing strong agreement between CL values and visual reading for all four methodological variants.
